# A rare case of intestinal internal hernia beneath the external iliac artery following radical cystectomy

**DOI:** 10.1093/jscr/rjad118

**Published:** 2023-03-18

**Authors:** Lachlan Allan, Dinushi Perera, Richard Curran

**Affiliations:** General Surgery, Westmead Hospital, Hawkesbury Rd, Westmead, NSW 2145, Australia; General Surgery, Westmead Hospital, Hawkesbury Rd, Westmead, NSW 2145, Australia; General Surgery, Westmead Hospital, Hawkesbury Rd, Westmead, NSW 2145, Australia

**Keywords:** Small bowel obstruction, intestinal ischaemia, internal hernia, external iliac artery, iatrogenic arterial injury

## Abstract

Gastrointestinal complications following radical cystectomy (RC) are a common occurrence, with small bowel obstruction (SBO) a known complication. Limited cases have been reported of SBO following RC due to internal herniation of the small intestine around the ureter, ileal conduit, obturator nerve and, as a consequence of retroperitoneal lymphadenectomy, even the abdominal vasculature. We present a rare case in which intestinal herniation beneath the external iliac artery (EIA) resulted in a closed-loop SBO with ischaemia and necrosis. Intra-operative transection of the unrecognised EIA occurred, necessitating primary arterial repair. This case highlights the importance of maintaining a high index of suspicion for complex pathology and anatomical variations in patients following RC and other operations involving retroperitoneal lymphadenectomy.

## INTRODUCTION

After abdominal surgery, the most common cause of small bowel obstruction (SBO) are adhesions [1]. Internal hernia represent a small portion of post-operative SBO, however can lead to closed-loop obstruction, intestinal ischaemia, necrosis and perforation. There have been reports of internal herniation of the small bowel around the ureter, ileal conduit and obturator nerve following radical cystectomy (RC) [2–4]. In addition, SBO secondary to internal herniation around the major abdominal vasculature is an extremely rare life and limb threatening pathology that has been described following RC and pelvic lymphadenectomy [5, 6].

We present a case of internal herniation of the small bowel beneath the left external iliac artery (EIA) causing SBO and ischemia following RC and ileal conduit formation, complicated by intra-operative iatrogenic EIA injury. To the author’s knowledge, this is the only reported case of SBO caused by internal hernia associated with major abdominal vessels complicated by iatrogenic injury to the involved vessel.

## CASE REPORT

**Figure 1:**
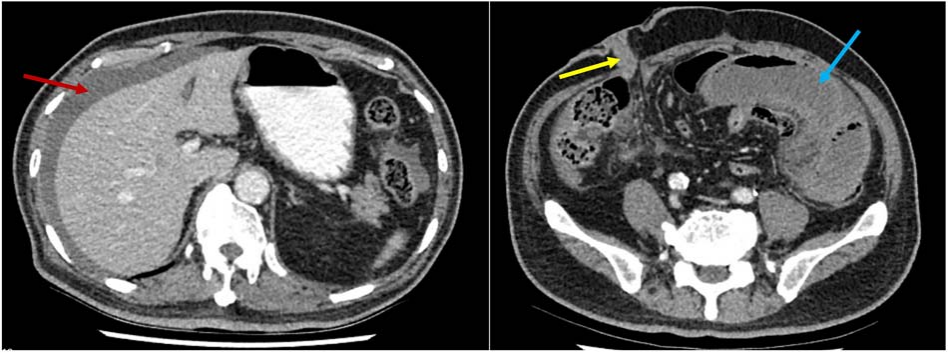
CT scan axial slices demonstrating perihepatic free fluid (red arrow) and dilated loops of small bowel in the left iliac fossa (blue arrow). The ileal conduit (yellow arrow) in the right iliac fossa appears healthy.

A 75-year-old male presented to our institution 4 months after RC for bladder cancer with acute abdominal pain and vomiting. Vital signs were within normal limits, the abdomen was distended and tender in the left lower quadrant. Significant biochemical derangements with metabolic acidosis and elevated lactate were demonstrated. A computed tomography (CT) scan of the abdomen and pelvis demonstrated a closed-loop SBO in the left lower quadrant with intrabdominal free fluid (Fig. 1). The right-sided ileal conduit appeared healthy.

Emergent laparotomy revealed an ischaemic segment of ileum in the left lower quadrant and haemorrhagic peritonitis. The closed-loop obstruction was adherent to the pelvic brim with a transition point identified at a firm, narrow, tubular structure that prevented evisceration of the ischaemic segment. This was non-pulsatile and unable to be traced proximally or distally due to the surrounding dilated loops of small bowel. The structure was sharply divided to facilitate mobilisation and resection of the ischaemic bowel. Post-transection it was noted this was a vascular structure, presumed the left EIA, beneath which the small intestine had herniated causing obstruction. Examination demonstrated absence of left femoral and pedal pulses. Intra-operative consultation from Vascular Surgery confirmed an iatrogenic injury to the left EIA. Thrombus was removed from both proximal and distal ends of the EIA which was subsequently primarily repaired with 5–0 prolene suture (Ethicon inc., Somerville, NJ, USA). The peritoneum was closed over the arterial anastomosis. The necrotic bowel was resected, and a side-to-side functional end-to-end anastomosis was fashioned with an nTLC 75 linear stapler (Ethicon inc.) and the entero-enterostomy was closed with a TX60 stapler (Ethicon inc.). The ileal conduit remained healthy and urine output was noted at the end of the operation.

**Figure 2:**
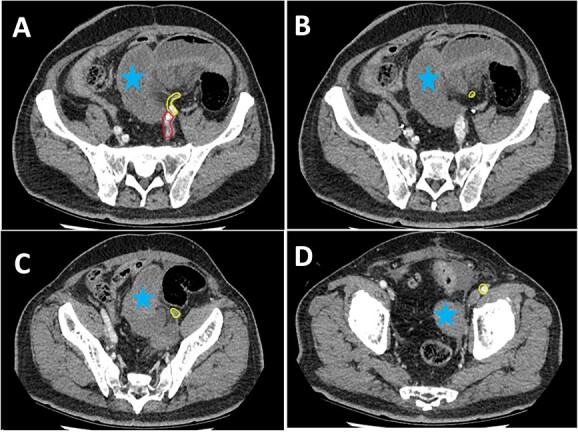
Series of axial images of initial CT progressing from most superior (image A) to inferior (image D). The EIA has been highlighted in yellow from immediately inferior to the bifurcation of the common iliac artery. The internal iliac artery can be seen in red in image **A**. Images **B** and **C** demonstrate a collapsed EIA between dilated loops of small bowel suggesting involvement in the internal hernia. Image **D** demonstrates opacification of the EIA indicating flow distal to the internal hernia. Dilated loops of bowel have been marked with a blue star.

Histopathological examination confirmed ischaemic enteritis with patchy mural necrosis. Post-operative CT demonstrated healthy small bowel and patent left EIA (Figs 2 and 4). The patient recovered well and was discharged after a short hospital admission, with no ongoing abdominal symptoms or lower limb claudication. Follow up in the community was unremarkable with no further interventions required.

**Figure 3:**
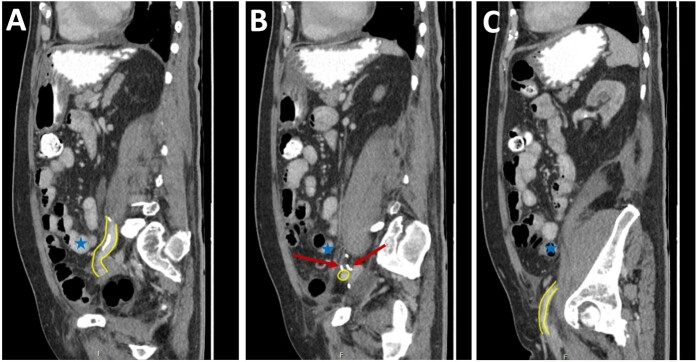
Post-operative CT scan. Series of sagittal images progressing from most medial (image **A**) to most lateral (image **C**). The patent EIA has been highlighted in yellow. Surgical clips are noted in image **B** (red arrow). Normal small bowel is noted in all images (blue stars).

**Figure 4:**
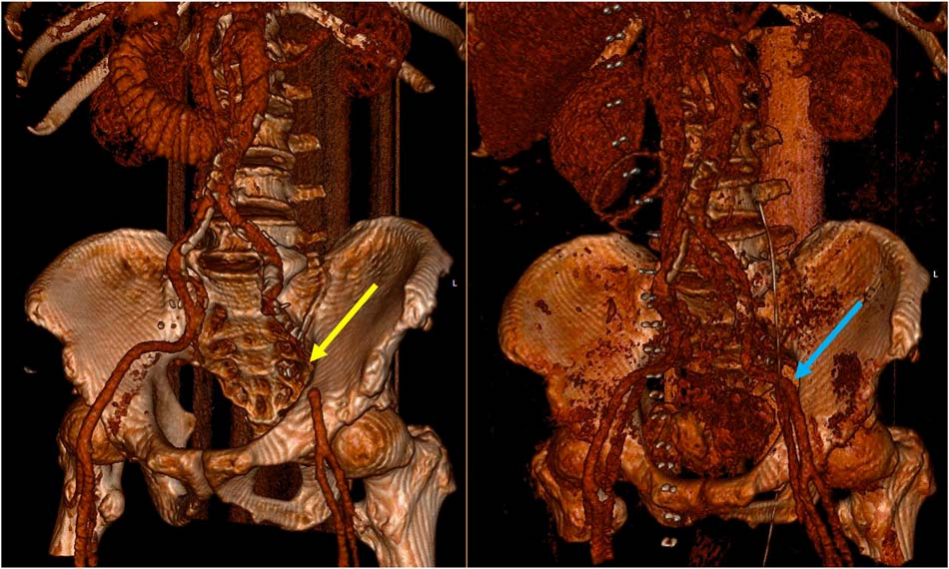
Three-dimensional reconstruction of the pre-operative (left) and post-operative (right) CT scan. The pre-operative CT demonstrates a segment of EIA that cannot be reconstructed due to the slow flow through the segment involved in the SBO (yellow arrow). The post-operative image demonstrates a patent EIA following vascular repair (blue arrow).

## DISCUSSION

RC is associated with high rates of morbidity and mortality. A total of 29% of complications following surgery are of the gastrointestinal (GI) system with the majority of these being small bowel ileus [7]. SBO comprises 4–5% of GI complications following RC and is predominantly adhesional. Internal herniation causing intestinal obstruction is an uncommon entity, accounting for 0.5–4.1% of acute SBO [8, 9]. Operative intervention is required in cases where closed-loop obstruction, failure of medical management or complications of intestinal obstruction are observed.

Internal herniation related to the external iliac arteries is an extremely rare pathological process. Fewer than 10 cases have been described following radical lymphadenectomy for testicular, prostate and gynaecological tumours [9–11]. To our knowledge, there are only two other reported cases of this occurrence following RC [5, 6]. The process of mobilisation of the vasculature from the retroperitoneum creates a potential space for intestinal herniation. The authors of some of these reports advocate for consideration of closure of the potential space through primary peritoneal repair or peritoneal grafting, however, this remains controversial [10–12]. Skeletonisation of the abdominal vasculature is a key operative step in RC as it improves oncological outcomes [9], however. there is limited literature discussing the role of retroperitonealisation of the vasculature following skeletonisation in RC.

Pre-operative identification of internal hernias would allow for optimal management and planning, however diagnosis of intestinal herniation causing SBO on imaging can be challenging. CT features such as atypical bowel configuration, mesenteric abnormalities (including displacement, twisting or stretching of mesenteric vessels) and the position of surrounding viscera raise the index of suspicion of internal herniation [13]. In this case, retrospective review of the CT scan demonstrates the proximity and compression or partial occlusion of the segment of EIA within the closed-loop obstruction (Figs 3 and 4). This is concordant with our intra-operative findings of a narrow, non-pulsatile structure.

Intra-operative signs of internal herniation related to vascular structures can be atypical. In our patient, the EIA was firm, collapsed and not pulsatile. In most other reported cases the bowel was able to be reduced, a long segment of skeletonised artery was visible or authors were able to identify a pulsation or thrill. Our case highlights that these findings may not always be present. Furthermore, we demonstrate use of sharp rather than diathermy division and early involvement of a Vascular Surgeon for assessment and repair can prevent complications from the injury.

We present a unique case in which intestinal herniation beneath the left EIA caused an SBO complicated by intestinal ischaemia. The rarity of this pathological process alongside the atypical radiological and intra-operative findings contributed to an iatrogenic arterial injury occurring. We highlight the importance of considering atypical causes of GI complications and maintaining a high index of suspicion for post-surgical pathologies aside from adhesions in patients who have undergone RC and other operations with retroperitoneal dissection.

## CONFLICT OF INTEREST STATEMENT

None declared.

## FUNDING

None.

## DATA AVAILABILITY

Data sharing is not applicable to this article as no new data were created or analyzed in this study.
